# Insights Into Intra-arterial Thrombolysis in the Modern Era of Mechanical Thrombectomy

**DOI:** 10.3389/fneur.2019.01195

**Published:** 2019-11-13

**Authors:** Alicia C. Castonguay, Mouhammad A. Jumaa, Osama O. Zaidat, Diogo C. Haussen, Ashutosh Jadhav, Hisham Salahuddin, Syed F. Zaidi

**Affiliations:** ^1^Department of Neurology, The University of Toledo Health Science Campus, Toledo, OH, United States; ^2^St. Vincent Mercy Hospital, Toledo, OH, United States; ^3^Department of Neurology and Neurosurgery, Marcus Stroke and Neuroscience Center, Grady Memorial Hospital, Emory University School of Medicine, Atlanta, GA, United States; ^4^Department of Neurology, University of Pittsburgh Medical Center, Pittsburgh, PA, United States

**Keywords:** intra-arterial, thrombolysis, thrombectomy, stroke, occlusion

## Abstract

**Background and Purpose:** The role of intra-arterial (IA) thrombolysis in modern endovascular therapy is not well-understood. Here, we surveyed neurointerventionalists to understand their current clinical practices and opinions of IA thrombolysis in the new era of mechanical thrombectomy (MT).

**Method:** A 24-question anonymous survey was distributed via email to the members of the Society of Vascular and Interventional Neurology.

**Results:** One hundred and four responses were included in the analysis. Most respondents were interventional neurologists (76.9%) and had ≥5-years in neuro-interventional practice (80.8%). IA thrombolytics are presently used by 60.6%. Aspiration plus stent-retriever was the most common MT approach used with IA-thrombolysis (66.0%). IA-thrombolysis was used in mainly three approaches: (1) treatment of primary distal occlusions, (2) as rescue after proximal occlusion thrombectomy, and (3) or as adjunct therapy to primary MT approach. The most frequent IA-rtPA dose was 3–10 mg, with 1 mg/min infusion rate (56.6%). 84.9% do not have a standardized protocol for administering IA-rtPA. About half (50.9%) believed there should be no time limit for administering IA lytic if there is a favorable imaging profile, while 30.2% indicated ≤6 h. Most respondents (76.5%) would consider using IA-tenecteplase in a trial setting. Only 12.9% felt there was no role for IA thrombolysis in modern endovascular practice. Respondents with ≥10-years' experience were less supportive of the future of IA lytic (98.0 vs. 76.4%, *p* = 0.006).

**Conclusion:** IA-thrombolysis is currently used in clinical practice; however, there is no clear consensus on best practices or criteria for administration. Further studies are needed to define the role of IA-thrombolysis in the context of MT.

## Introduction

Although mechanical thrombectomy (MT) has become the gold standard for the treatment of large vessels occlusions (LVOs), limited complete reperfusion rates by current generation stent-retrievers give way for adjunctive therapies to potentially augment their revascularization effectiveness. Complete or near-complete rates of reperfusion correlate with better functional outcomes in acute ischemic stroke patients (1).

Intra-arterial (IA) thrombolysis, once a first-line therapy for LVOs prior to the advent of MT, has reemerged with a potential new role in the modern endovascular era. Recent studies have demonstrated promising results of IA recombinant tissue plasminogen activator (rtPA) in the context of MT (2–4); however, the role of IA thrombolysis in contemporary endovascular therapy is not well-understood and limited data exists on its current use in real-world practice. Here, we surveyed the neuro-interventional field to evaluate the current clinical practices and opinions of IA thrombolysis in the context of MT and to better understand its future role in endovascular stroke therapy.

## Methods

A 24-question survey was developed to understand current practices and opinions of physicians on the use of IA thrombolytics in endovascular stroke therapy. The survey was designed using SurveyMonkey (SurveyMonkey, Inc., San Mateo, CA, www.surveymonkey.com), an online survey development cloud-based software. This survey was classified as exempt human subject research by the University of Toledo institutional review board. As such, written informed consent was not required. The survey link was distributed to the Society of Vascular and Interventional Neurology members (SVIN) via email. All responses were anonymous, and the survey link allowed for only one completion per individual. Questions were presented in the following categories: (1) clinical and practice background, (2) IA thrombolytic use, (3) case examples, and (4) IA thrombolysis in modern endovascular practice (Table 1). Skip logic was incorporated to promote efficiency and ease of survey navigation. For analysis, all survey data were presented as frequencies of responses.

**Table 1:** Intra-arterial thrombolysis survey questions and responses.

**Questions**	**Answers**
**Clinical and practice background**	
1. What is your clinical background?	Interventional neurologist (80/104; 76.9%) Endovascular Neurosurgeon (8/104; 7.7%) Interventional Neuro-radiologist (16/104; 15.4%)
2. How long have you been in neuro-interventional practice?	0–4 years (20/104; 19.2%) 5–9 years (43/104; 41.3%) ≥10 years (41/104; 39.4%)
3. What is the setup of your neuro-interventional practice?	Academic (51/104; 49.0%) Private-academic (with residents and/or fellows) (29/104; 27.9%) Private (no fellows or residents) (24/104; 23.1%)
4. What is your center's certification status?	JC Comprehensive Stroke Center (55/104; 52.9%) DNV Comprehensive Stroke Center (25/104; 24.0%) Primary Stroke Center with interventional capabilities (14/104; 13.5%) Certified Thrombectomy Ready Hospital (10/104; 9.6%)
5. On average, how many mechanical thrombectomies do you perform per year?	0–23 per year (14/104; 13.5%) 24–47 per year (23/104; 22.1%) 48–74 per year (23/104; 22.1%) ≥75 per year (44/104; 42.3%)
6.Were you a participating investigator in IMSIII and/or PROACT II?	Yes (24/104; 23.1%) No (80/104; 76.9%)
**IA thrombolytic use**	
7. How often do you use IA thrombolytic for treatment for acute ischemic stroke?	Never (41/104; 39.4%) 1–5 cases per year (49/104; 47.1%) 6–10 cases per year (6/104; 5.8%) 11–20 cases per year (4/104; 3.8%) >20 cases per year (4/104; 3.8%)
8. How is IA rt-PA used in your practice? (check all that apply)	Primary therapy alone for the target occlusion (similar to PROACT II or MELT trials) (2/53; 3.8%) Primary distal occlusion (M3/4, A2, P2, etc) (22/53; 41.5%) Rescue therapy to the primary target occlusion that failed mechanical thrombectomy (19/53; 35.9%) Rescue therapy for unsatisfactory results of the primary modality to address distal embolization (34/53; 64.2%) Rescue therapy for unsatisfactory results of the primary modality to address embolization into new territory (28/53; 52.8%) Adjunctive therapy in conjunction with mechanical thrombectomy from the beginning of the procedure (as an add-on and not to address failed MT or unsatisfactory results) (3/53; 5.7%)
9. What is the average dosage of IA rt-PA administered for large vessel occlusions of the anterior circulation?	0–2 mg (5/53; 9.4%) 3–5 mg (19/53; 35.9%) 6–10 mg (17/53; 32.1%) 11–15 mg (5/53; 9.4%) 16–20 mg (4/53; 7.6%) >20 mg (0/53; 0%) I do not use IA rt-PA for anterior circulation LVOs (3/53; 5.7%)
10. In the case of distal M3/M4 occlusion, where would you generally administer IA rt-PA?	Within the clot itself (14/53; 26.4%) Within the occluded branch proximal to the clot (33/53; 62.3%) Distal MCA M1 (3/53; 5.7%) Proximal MCA (0/53; 0%) ICA (0/53; 0%) I do not use IA rt-PA to treat distal occlusions (3/53; 5.7%)
11. In your practice, what is the average dose of IA rt-PA administered for distal M3 or distal A2 occlusions?	0–2 mg (7/53; 13.2%) 3–5 mg (23/53; 43.4%) 6–10 mg (15/53; 28.3%) 11–15 mg (4/53; 7.6%) 16–20 mg (1/53; 1.9%) >20 mg (0/53; 0%) I do not use IA rt-PA for distal occlusions (3/53; 5.7%)
12. If you encounter a tandem lesion, what would be your opinion about giving IA rt-PA?	Never use IA rt-PA for a tandem lesion (23/53; 43.4%) Only use IA rt-PA if performing angioplasty only (8/53; 15.1%) It doesn't matter as long as no dual anti-platelets are given PO or IV GP IIb/IIIa (6/53; 11.3%) I would use IA rt-PA regardless of the tandem lesion treatment approach (16/53; 30.2%)
13. Do you have a standardized protocol for giving IA rt-PA?	Yes (8/53; 15.1%) No (45/53; 84.9%)
14. In general, how long do you infuse IA rt-PA?	5 min/1 mg (10/53; 18.9%) 2 min/1 mg (11/53; 20.8%) 1 min/1 mg (30/53; 56.6%) Other (2/53; 3.8%)
15. When using IA rt-PA, do you also use microwire maceration?	Yes, sometimes (29/53; 54.7%) Yes, I always use if technically feasible (5/53; 9.4%) No (19/53; 35.9%)
16. Which type(s) of mechanical thrombectomy do you use with IA rt-PA? (check all that apply)	Aspiration only (17/53; 32.1%) Aspiration plus stent-retriever (35/53; 66.0%) Stent-retriever only (14/53; 26.4%) None, I would use IA rtPA alone (11/53; 20.8%)
17. Check which scenario(s) you would NOT feel comfortable giving a patient IA rt-PA (check all that apply)	Age more than 85 (9/53; 17.0%) NIHSS >25 (8/53; 15.1%) IV rt-PA prior to endovascular therapy (20/53; 37.7%) None of the above (26/53; 49.1%)
18. What would be your ASPECTS cut off for use of IA rt-PA during MT?	8–10 (11/53; 20.8%) 7–10 (9/53; 17.0%) 6–10 (19/53; 35.9%) No cut off (14/53; 26.4%)
19. What would be your core infarct volume (using MR perfusion or CT perfusion) cut off for NOT giving IA rt-PA?	0–25 ml (7/53; 13.2%) 26–50 ml (8/53; 15.1%) 51–70 ml (17/53; 32.1%) 71–100 ml (15/53; 28.3%) >100 ml (6/53; 11.3%)
20. IA thrombolysis should be administered in which time window?	≤6 h (16/53; 30.2%) ≤8 h (7/53; 13.2%) ≤12 h (0/53; 0%) ≤16 h (0/53; 0%) ≤24 h (3/53; 5.7%) No time limit with favorable imaging profile (27/53; 50.9%)
**Case Examples**	
21. For case example #1, what would be your preferred treatment approach?	No further treatment (19/90; 21.1%) Aspiration only (16/90; 17.8%) Stent-retriever only (14/90; 15.6%) Aspiration + stent-retriever (18/90; 20.0%) IA rt-PA only (23/90; 25.6%)
22. For case example #2, what would be your preferred treatment approach?	No further treatment (41/87; 47.1%) Aspiration only (3/87; 3.5%) Stent-retriever only (4/87; 4.6%) Aspiration + stent-retriever (3/87; 3.5%) IA rt-PA only (36/87; 41.4%)
**IA Thrombolysis in Modern Endovascular Practice**	
23. In a trial setting, would you consider using IA tenecteplase (TNK) for treatment of distal occlusions?	Yes (65/85; 76.5%) No (7/85; 8.2%) Maybe (13/85; 15.3%)
24. I believe that IA thrombolysis has a role in modern endovascular practice?	Yes, IA thrombolysis has a role in modern endovascular practice (32/85; 37.6%) No, IA thrombolysis does not have a role in modern endovascular practice. (11/85; 12.9%) Maybe, more evidence is needed to clarify the role of IA thrombolysis in modern endovascular practice. (42/85; 49.4%)

## Results

From February to May 2019, 106 responses were collected with an 80% completion rate. Of those, 104 respondents had completed at minimum questions 1 through 7 and were included in this analysis (Supplementary Table 1).

### Clinical and Practice Background

Of the 104 respondents, 80 (76.9%) were interventional neurologists, 16 (15.4%) interventional neuro-radiologists, and 8 (7.7%) were endovascular neurosurgeons (Supplementary Table 1). Most respondents had ≥5-years of neuro-interventional experience (80.8%). Almost half (49.0%) were in an academic practice, and 52.9% were in a Joint Commission certified comprehensive stroke center. On average, 42.3% performed more than 75 MTs per year. Only 23.1% had previous experience as a participating investigator in the Interventional Management of Stroke-III and/or the Prolyse in Acute Cerebral Thromboembolism II trial.

### IA Thrombolytic Use

Most respondents (60.6%) used IA thrombolytics in their practice, of which 47.1% treated 1–5 cases/year with IA-rtPA (Table 1).

#### How IA Lytic Is Used

Of those that use IA lytics, 60.4% indicated that they used IA-rtPA in all three different approaches: (1) for treating primary distal occlusion, (2) as rescue therapy, and/or (3) adjunctive therapy (Supplementary Figure 1).

#### Dosage and Location of Infusion

The average IA-rt-PA dosage was 3–5 mg among 35.9% and 6–10 mg among 32.1% of respondents (Table 1). 56.6% infuse IA-rtPA at a rate of 1 mg/min and 54.7% sometimes use microwire clot maceration with IA-rtPA.

In reference to where in relation to the clot should IA lytic be administered for distal M3/M4 occlusions, the majority of respondents (62.3%) would administer IA-rtPA proximal to the clot and 26.4% would administer within the clot itself (Table 1).

#### MT Approaches

When questioned regarding types of MT that were used in conjunction with IA-rt-PA (irrespective of the approach), 70.0% chose a single response, of which 51.4% answered aspiration plus stent-retriever (Table 1, Supplementary Figure 2).

#### IA Lytic Criteria

Most respondents (86.8%) chose a single response when asked which scenario they would not feel comfortable giving a patient IA-rtPA, with 56.5% indicating that they would feel comfortable in all scenarios (age >85 years, NIHSS >25, and IV rt-PA prior to MT) and 32.6% would not feel comfortable using IA-rtPA in patients treated with IV-rtPA (Supplementary Table 1, Supplementary Figure 3).

There was no consensus by respondents on an Alberta Stroke Program Early CT Score cut-off for use of IA-rtPA, with 20.8% answering 8–10, 17.0% 7–10, 35.9% 6–10, and 26.4% preferred no cut-off. The core infarct volume (on MR/CT perfusion) cut-off for not giving IA-rtPA varied; however, 60.4% answered either 51–70 ml (32.1%) or 71–100 ml (28.3%).

Approximately half of the respondents feel that there should be no time limit for IA lytic administration with favorable imaging, while 30.2% indicted ≤6 h.

Importantly, 84.9% did not have a standardized protocol for administering IA-rtPA.

### Case Presentations

Two case examples were provided to gauge respondents preferred treatment approaches for distal occlusions after MT (Supplementary Table 1).

#### Distal Embolization, Post-MT, and IV-rtPA

**Figure 1:**
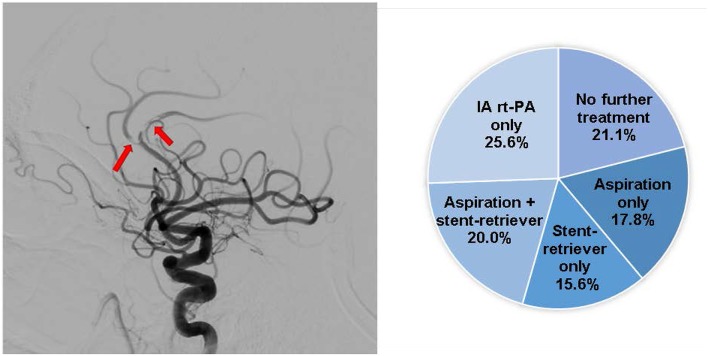
Case presentation **#**1. A 75-year-old with RICA T occlusions post-IV t-PA and MT with distal embolization into the right ACA A2. The MCA territory has completely recanalized; however, there is a subocclusive thrombus in the right pericallosal and callosmarginal divisions. Pie chart depicts the respondents' treatment preferences.

For case example 1 (Figure 1), treatment preferences varied; however, the majority of respondents agreed that further treatment is necessary (78.9%).

#### Distal Embolization, Post-MT Without IV-tPA

**Figure 2:**
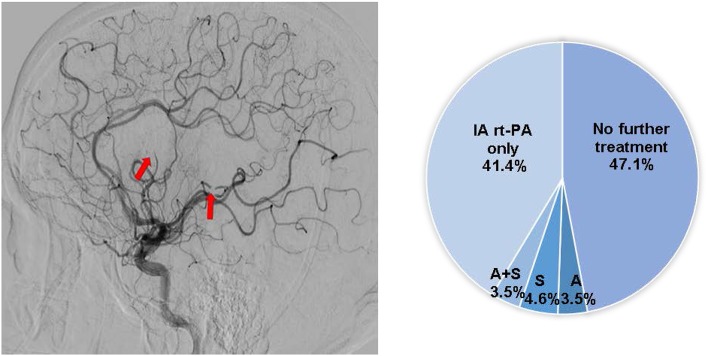
Case presentation #2. A 65-year-old with right sided weakness and aphasia, NIHSS 18, last known well 8 h prior to presentation, ASPECTS 9, with a left M1 occlusion on head CTA. Post left MCA MT, distal embolization occurred into multiple M3 branches. Pie chart depicts the respondents' treatment preferences. A, aspiration only; S, stent-retriever only; A+S, aspiration and stent-retriever.

For case example 2 (Figure 2), about half of the respondents (47.1%) would recommend no further treatment and 41.4% would treat with IA-rtPA only.

### IA Thrombolysis in Modern Endovascular Practice

#### New IA Lytics

When asked if respondents would consider using IA tenecteplase for treatment of distal occlusions in a trial setting, 76.5% answered yes (Supplementary Table 1).

#### Future of IA Thrombolysis

Almost half (49.4%) of those surveyed believed that IA thrombolysis may have a role in modern endovascular practice, but more evidence is needed, while 37.6% agreed that IA thrombolysis has a role in current endovascular practice (Supplementary Table 1).

### Impact of Case Volume and Experience

**Figure 3:**
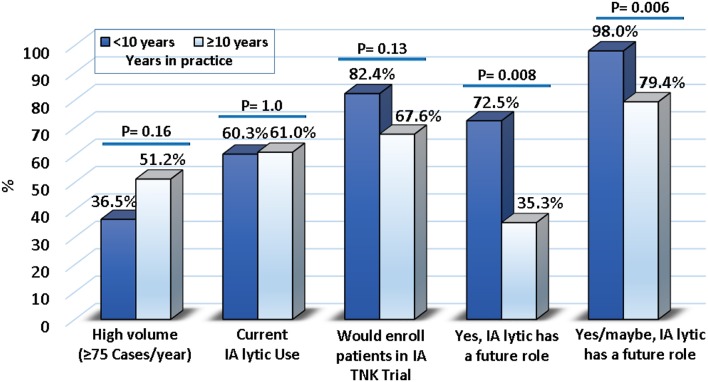
Results stratified by years of experience.

When stratifying the results by case volume or experience level, no difference was found in use of IA lytic; however, those with ≥10 years of experience were less enthusiastic about the future of IA lytic (*p* = 0.006) (Figure 3, Supplementary Tables 1, 2).

## Discussion

To date, little is known about the use of IA thrombolysis in the current era of endovascular stroke therapy. Recent studies after the landmark MT trials (5–10) have investigated IA-rtPA use in conjunction with MT (2–4); however, these studies were limited by their retrospective non-randomized nature, small sample size, and heterogeneous populations and techniques. Our study revealed that most respondents use IA thrombolysis in their current clinical practice; however, few implement standardized protocols for IA-rtPA administration.

Our survey highlights the variability of IA-rt-PA use, with most respondents using IA lytic for primary distal occlusions, or RT for distal embolization and embolization into new territory after MT, and in different settings of MT, including aspiration and stent-retrievers. When presented with two different cases of distal occlusions after MT, there was no consensus on the preferred treatment approach. As distal vessel occlusions (DVO) may cause significant deficits depending on the eloquence of the affected branch or branches (11, 12), endovascular therapies, such as IA rt-PA, may be viable treatment options for these occlusions. A recent retrospective single center case study of DVO demonstrated an acceptable safety and reperfusion profile with endovascular therapy (52% treated with IA-rtPA) (13). Although newer devices have made MT in distal vessels possible, in cases of extreme tortuosity, IA thrombolytics may be preferable.

Additionally, we also found a wide-range of IA-rtPA dosing for anterior circulation LVOs and distal occlusions; however, more than half of survey respondents administered in the range of 3–10 mg with varying infusion rates. Recent MT trials, MR CLEAN, and ESCAPE, allowed the use of IA-rtPA (9, 10). MR CLEAN allowed for a maximum of dose of 90 mg (if IV-rtPA was not administered) and 30 mg (if IV-rtPA was administered) administered via micro-catheter at the level of occlusion. The ESCAPE Trial protocol recommended a maximum dose of 10 mg rtPA via micro-catheter for use as adjunctive therapy. Two retrospective studies reported dosing of IA rt-PA of up to 15 mg and <5 mg (3, 4). As there are no standardized dosing guidelines, prospective studies are warranted to determine the dosing threshold for IA-rtPA.

Currently, there are no established criteria for IA thrombolysis administration in the context of MT and results of our survey highlight the lack of consensus among MT practitioners. Less than 50% of respondents would feel comfortable administering IA-rtPA to those >85 years, NIHSS>25, or received IV-rt-PA prior to endovascular therapy and no clear consensus was reached for a cut-off regarding Alberta Stroke Program Early CT Score or time window. The above results further demonstrate the need for clinical studies to define the criteria for use of IA thrombolysis in endovascular therapy, including the optimal patient population.

### Future of IA Thrombolysis

Respondents believed that IA thrombolysis may have a place in modern endovascular practice, but more evidence is required to define its role. This was supported by the respondents' enthusiasm for enrolling in future IA lytic trials, including IA tenecteplase.

### Limitations

Our study has several limitations, including the inherent limitations of survey research. As this survey was administered to SVIN members, results may not be inclusive of the practice patterns or opinions of other MT practitioners not affiliated with SVIN. For example, 76.9% of respondents were interventional neurologists and only 7.7% were endovascular neurosurgeons. As not all respondents answered all survey questions, it is possible that lack of response may have introduced selection bias into the survey results. Additionally, the small sample size may limit the interpretability and generalizability of this study.

## Conclusion

Our survey demonstrates that IA thrombolysis is widely used in current practice. These results support the need for further studies on IA thrombolysis in the context of MT and may serve as a guide for the design of future IA thrombolysis clinical studies.

## Data Availability Statement

All datasets generated for this study are included in the article/Supplementary Material.

## Ethics Statement

This survey was classified as exempt human subject research by the University of Toledo institutional review board. As such, written informed consent was not required.

## Author Contributions

AC, SZ, OZ, DH, MJ, and AJ participated in the design, analysis, drafting, and editing of the manuscript. HS participated in the analysis and critical review of the manuscript.

### Conflict of Interest

The authors declare that the research was conducted in the absence of any commercial or financial relationships that could be construed as a potential conflict of interest.
