# Supplementary material for: Insights Into Intra-arterial Thrombolysis in the Modern Era of Mechanical Thrombectomy
Source: Front Neurol. 2019 Nov 13;10:1195. doi: 10.3389/fneur.2019.01195 (PMC6863970; doi:10.3389/fneur.2019.01195)
Supplement: Supplementary file 1 [file Table_1.DOCX]

**SUPPLEMENTAL MATERIAL**

**Supplementary Table I: Selected survey results stratified by case volume (≥75 cases versus <75 cases per year).**

|  | **≥75 cases (N,%)** | **<75 Cases (N,%)** | **P-value** |
| --- | --- | --- | --- |
| **Current IA Lytic Use (N=44) (N=60)** | | | |
| Never | 15 (34.1) | 26(43.3) | 0.42 |
| 1-5 cases | 22 (50.0) | 27 (45.0) | 0.69 |
| 6-10 cases | 1 (2.3) | 5 (8.3) | 0.4 |
| 11-20 cases | 4 (9.9) | 0 (0) | **0.03** |
| >20 cases | 2 (4.5) | 2 (3.3) | 1.0 |
| **IA-TNK Trial (N=37) (N=48)** | | | |
| Yes | 30 (81.1) | 35 (72.9) | 0.46 |
| No | 1 (2.7) | 6 (12.5) | 0.13 |
| Maybe | 6 (16.2) | 7 (14.6) | 1.0 |
| **Future of IA Lytic? (N=37) (N=48)** | | | |
| Yes | 16 (43.2) | 16 (33.3) | 0.38 |
| No | 5 (13.5) | 6 (12.5) | 1.0 |
| Maybe | 16 (43.2) | 26 (54.2) | 0.38 |

**IA, intra-arterial; TNK, tenecteplase**

**Supplementary Table II: Selected survey results stratified by years in neuro-interventional practice (<10 years versus ≥10 years).**

|  | **<10 years (N,%)** | **≥10 years (N,%)** | **P-value** |
| --- | --- | --- | --- |
| **Case volume (N=63) (N=41)** | | | |
| ≥75 cases | 23 (36.5) | 21 (51.2) | 0.16 |
| <75 cases | 40 (63.5) | 20 (48.8) | 0.16 |
| **Current IA Lytic Use (N=63) (N=41)** | | | |
| Never | 25 (39.7) | 16 (39.0) | 1.0 |
| 1-5 cases | 32 (50.8) | 17 (41.5) | 0.42 |
| 6-10 cases | 3 (4.8) | 3 (7.3) | 0.68 |
| 11-20 cases | 1 (1.6) | 3 (7.3) | 0.30 |
| >20 cases | 2 (3.2) | 2 (4.9) | 0.65 |
| **IA-TNK Trial (N=51) (N=34)** | | | |
| Yes | 42 (82.4) | 23 (67.6) | 0.13 |
| No | 2 (3.9) | 5 (14.7) | 0.11 |
| Maybe | 7 (13.7) | 6 (17.6) | 0.76 |
| **IA Lytic Future? (N=51) (N=34)** | | | |
| Yes | 37 (72.5) | 12 (35.3) | **0.008** |
| No | 1 (2.0) | 7 (20.6) | **0.006** |
| Maybe | 13 (25.4) | 15 (44.1) | 0.10 |
| Yes/Maybe | 50 (98.0) | 27 (79.4) | **0.006** |

**IA, intra-arterial; TNK, tenecteplase**

**SUPPLEMENTAL FIGURES**

**
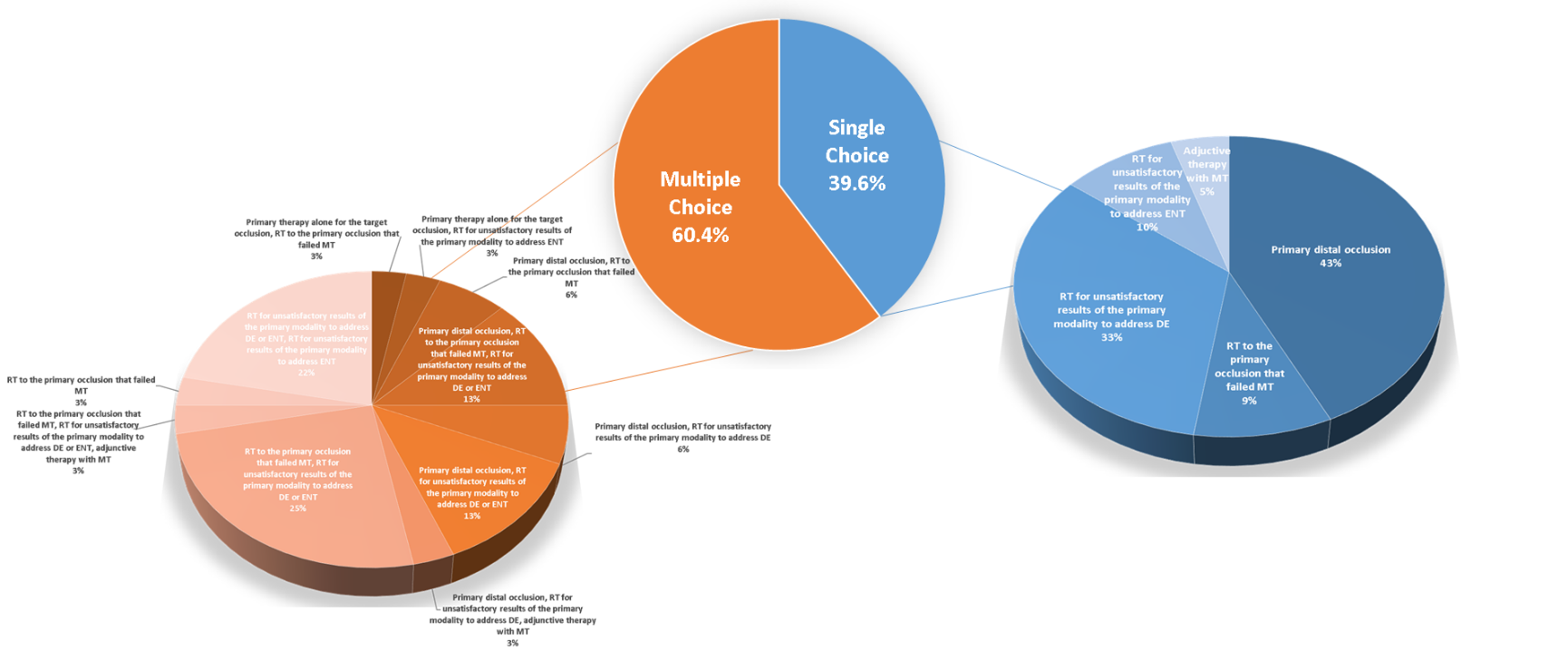
**

**Supplementary Figure I: IA rt-PA current practices.** Responses for how IA rtPA is used in current practices were collated as multiple choice or single choice (center pie chart), then further stratified by specific responses in each category for question 18. DE, distal embolization; ENT, embolization into new territory; MT, mechanical thrombectomy; RT, rescue therapy

**
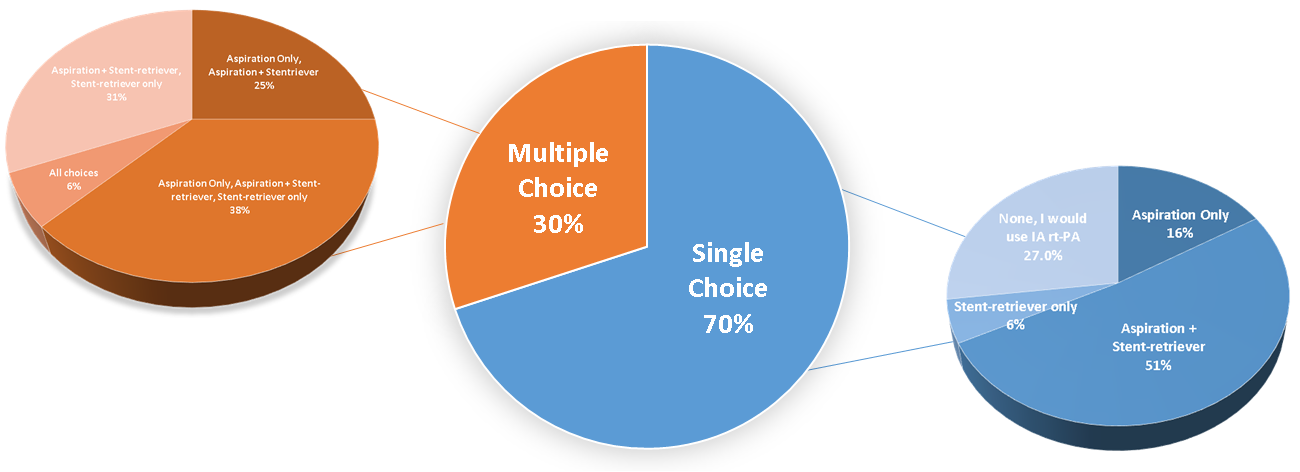
**

**Supplementary Figure II. Type (s) of mechanical thrombectomy used with IA rt-PA.** Responses for question 16 were collated as multiple choice or single choice (center pie chart), then further stratified by specific responses in each category for question 16.

**
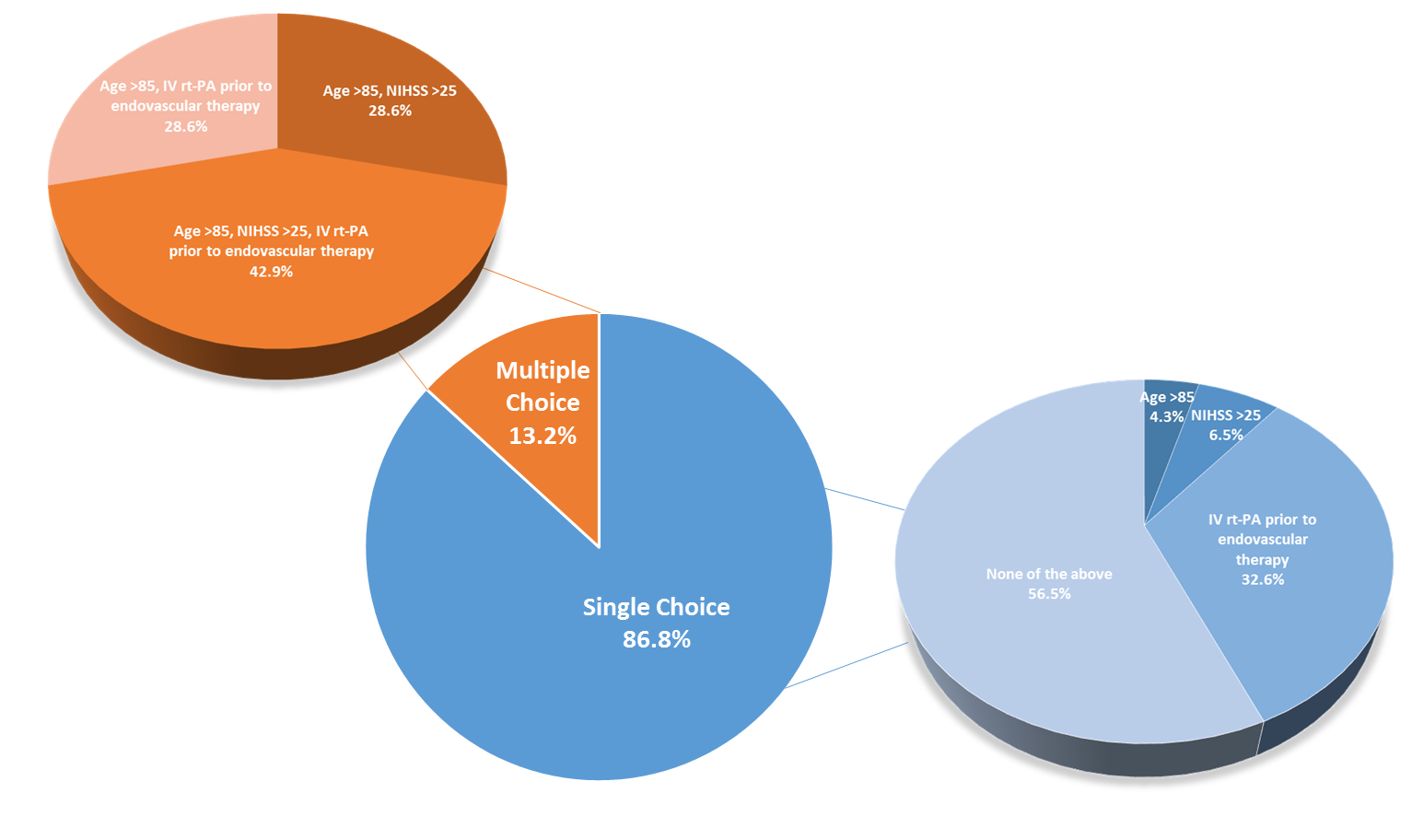
**

**Supplementary Figure III: Criteria for not administering IA rt-PA.** Responses for scenarios in which respondents would not feel comfortable using IA rt-PA were collated as multiple choice or single choice (center pie chart), then further stratified by specific responses in each category for question 17.
